# Tracking Progress Toward Polio Eradication — Worldwide, 2013–2014

**Published:** 2015-04-24

**Authors:** Kimberly A. Porter, Ousmane M. Diop, Cara C. Burns, Rudolph H. Tangermann, Steven G.F. Wassilak

**Affiliations:** 1 Global Immunization Division, CDC; 2 Polio Eradication Department, World Health Organization, Geneva, Switzerland; 3 Division of Viral Diseases, CDC

Global efforts to eradicate polio began in 1988 and have been successful in all but two of the six World Health Organization (WHO) regions. Within these two regions (African and Eastern Mediterranean), three countries (Afghanistan, Nigeria, and Pakistan) have never interrupted transmission of wild poliovirus (WPV) (1). Outbreaks following importation of WPV from these countries occurred in the Horn of Africa (2), Central Africa, and in the Middle East during 2013–2014 (3). The primary means of tracking polio is surveillance for cases of acute flaccid paralysis (AFP), the main symptom of polio, followed by testing of AFP patients’ stool specimens for both WPV and vaccine-derived poliovirus (VDPV) in WHO-accredited laboratories within the Global Polio Laboratory Network (GPLN). This is supplemented with environmental surveillance (testing sewage for WPV and VDPV) (4). Both types of surveillance use genomic sequencing for characterization of poliovirus isolates to map poliovirus transmission and for identifying gaps in AFP surveillance by measuring genetic divergence between isolates. This report presents 2013 and 2014 poliovirus surveillance data, focusing primarily on the two WHO regions with endemic WPV transmission, and the 29 countries (African Region = 23; Eastern Mediterranean Region = six) with at least one case of WPV or circulating VDPV (cVDPV) reported during 2010–2014. In 2013, 20 of these 23 African region countries met both primary surveillance quality indicators; in 2014, the number decreased to 15. In 2013, five of the six Eastern Mediterranean Region countries met the primary indicators, and in 2014, all six did. To complete and certify polio eradication, surveillance gaps must be identified and surveillance activities, including supervision, monitoring, and specimen collection, further strengthened.

## Acute Flaccid Paralysis Surveillance

In all African Region countries, 20,547 AFP cases were reported in 2013. In 2014, this number of cases increased to 22,451. In 2013, a total of 80 WPV type 1 (WPV1) cases were identified in four countries (Cameroon, Ethiopia, Kenya, and Nigeria); in 2014, there were 17 WPV1 cases identified in four countries (Cameroon, Equatorial Guinea, Ethiopia, and Nigeria). Date of onset for the latest WPV1 case was July 24, 2014, in Nigeria. A total of 13 cVDPV type 2 (cVDPV2) cases were identified in 2013 in four countries (Cameroon, Chad, Niger, and Nigeria). In 2014, 33 cVDPV cases (32 cVDPV2, one cVDPV1) were identified in three countries (Madagascar, Nigeria, and South Sudan) (Table 1).

In all Eastern Mediterranean Region countries, 11,246 and 12,505 AFP cases were reported in 2013 and 2104, respectively. In 2013, 336 WPV1 cases were identified in four countries (Afghanistan, Pakistan, Somalia, and Syria). In 2014, 342 WPV1 cases were identified in five countries (Afghanistan, Iraq, Pakistan, Somalia, and Syria), with the majority of cases in Pakistan. In 2013, 53 cVDPV cases (52 cVDPV2, one cVDPV3) were identified in four countries (Afghanistan, Pakistan, Somalia, and Yemen). Only one country, Pakistan, had identified cases of cVDPV2 (21 cases) in 2014 (Table 1).

The quality of AFP surveillance is measured by two principal indicators. The first is the non-polio AFP rate (i.e., the number of cases of non-polio AFP in children aged <15 years per 100,000 person-years). This indicator is met if the non-polio AFP rate is ≥2, which is considered sufficiently sensitive to identify cases from circulating poliovirus. The second indicator is the percentage of stool specimens considered adequate (i.e., collection within 14 days of paralysis onset, 24–48 hours apart, and arrival at the laboratory in “good” condition). To meet this indicator, adequate stool specimens should be collected for ≥80% of AFP cases, which shows that surveillance in the area can effectively identify WPV and VDPV among individuals with AFP (5).

Surveillance indicators were calculated for the 29 African Region and Eastern Mediterranean Region countries that reported one or more WPV or cVDPV cases during 2010–2014. In 2013, 20 (87%) of 23 countries in the African Region met both national indicators; the three that did not meet both indicators were Equatorial Guinea, Gabon, and Senegal. In 2014, 15 (65%) of these 23 countries met both indicators; the eight that did not meet both indicators were Cameroon, Central African Republic, Equatorial Guinea, Ethiopia, Gabon, Liberia, Niger, and Senegal. In 2013, five (83%) of six Eastern Mediterranean Region countries met indicators at the national level (Syria did not meet either). In 2014, all six Eastern Mediterranean Region countries met both indicators (Table 1). Surveillance indicators at the national level masked important heterogeneity of surveillance performance at subnational levels (Figure).

## Environmental Surveillance

Sampling and testing of sewage complements AFP surveillance by identifying poliovirus transmission that might occur in the absence of detected AFP cases (4). Environmental surveillance has been established at an increasing number of sites in specific areas within Afghanistan, Nigeria, and Pakistan, the three WPV-endemic countries. The total number of sites in these countries increased from 21 at the end of 2011 to 83 at the time of this report. Environmental surveillance is also conducted in more than 20 countries without active WPV transmission.

At the time of this report, sampling in Afghanistan is conducted at 11 sites and WPV1 has been detected in samples collected in Helmand, Kandahar, and Nangarhar. In Nigeria, sampling is currently conducted at 36 sites in nine states and the Federal Capital Territory. In May 2014, WPV1 was isolated from one sewage sample in Kaduna. Continued transmission of cVDPV2 that emerged in 2005 and of cVDPV2 imported from Chad in 2013 was documented during 2014 from sewage samples collected in six states. In Pakistan, sampling is conducted at 36 sites in every province except the Federally Administered Tribal Areas. The proportion of sewage samples positive for WPV1 in Pakistan increased from 20% in 2013 to 35% in 2014.

## Global Polio Laboratory Network

The GPLN consists of 146 WHO-accredited poliovirus laboratories in all WHO regions. GPLN member laboratories follow standardized protocols to 1) isolate and identify poliovirus, 2) differentiate the three poliovirus serotypes, 3) characterize polioviruses as WPV, Sabin-like poliovirus, or VDPV by intratypic differentiation (ITD) (6), and 4) conduct genomic sequencing. Sequencing results are used to monitor pathways of poliovirus transmission by comparing the nucleotide sequence of the VP1 coding region of poliovirus isolates. To meet standard laboratory timeliness indicators for stool specimen processing, laboratories should report ≥80% of poliovirus isolation results within 14 days of specimen receipt, ≥80% of ITD results within 7 days of isolate receipt, and ≥80% of sequencing results within 7 days of identifying isolate intratype. The standard programmatic indicator combining field and laboratory performance is to report ITD results for ≥80% of isolates within 60 days of paralysis onset of AFP cases. This indicator takes into account the entire interval from paralysis onset to specimen testing (the Eastern Mediterranean Region uses a 45-day timeframe). The accuracy and quality of testing at GPLN member laboratories is monitored through an annual accreditation program of onsite reviews and proficiency testing.

During 2013–2014, GPLN laboratories met timeliness indicators for poliovirus isolation in five of six WHO regions in each year and reporting indicators for receipt-to-ITD results in five of six regions in 2013 and all regions in 2014 (Table 2). The overall timeliness indicator for onset-to-ITD results was met in all regions in both years. The GPLN tested 197,658 stool specimens in 2013 and 204,078 stool specimens in 2014. In 2013, 416 WPV isolates were detected from AFP case samples compared with 359 WPV isolates detected in 2014. In addition, cVDPV was detected from 66 AFP case samples in 2013, compared with 54 cVDPV isolates detected in 2014 (data as of February 25, 2015).

In 2013, the only WPV1 genotypes isolated were WEAF-B1 and SOAS genotypes. In 2013, WEAF-B1 WPVs were detected in five countries (Cameroon, Ethiopia, Kenya, Nigeria, and Somalia). In 2014, WEAF-B1 WPVs were detected in five countries (Cameroon, Equatorial Guinea, Ethiopia, Nigeria, and Somalia). In Ethiopia, Kenya, Nigeria, and Somalia, only one WPV1 cluster* was detected among AFP cases, whereas WPV1 belonging to a different cluster was detected in Cameroon and Equatorial-Guinea. The SOAS genotype of WPV1 has circulated intensively in Afghanistan and Pakistan and has also been detected in Iraq and Syria. WPV1 isolates of the same cluster found in Iraq and Syria were most closely linked to a WPV1 isolate detected in an environmental sample from Pakistan. This virus was also detected in environmental samples from Egypt and Israel (5).

When genomic sequencing of an isolate shows ≥1.5% nucleotide divergence in the VP1-coding region from previously identified poliovirus isolates (an “orphan”) this highlights prolonged undetected circulation and gaps in AFP surveillance. Sequence analysis indicates that, as in 2013, WPV1 and cVDPV cases were likely being missed by AFP surveillance in 2014. In 2014, orphan WPV1 isolates were detected in ten of 306 WPV1 cases reported from Pakistan, five of 28 WPV1 cases reported in Afghanistan, one of six WPV1 cases reported from Nigeria, and one of five WPV1 cases reported from Cameroon. During 2014, orphan cVDPV viruses were also detected in Nigeria and Pakistan.

### Discussion

WPV has not been detected in a person with AFP in an African Region or Eastern Mediterranean Region country on the African continent since August 2014 and no sewage sample has tested positive for WPV on the African continent in the countries and areas conducting environmental surveillance in almost 1 year. Although this is an encouraging finding, undetected circulation of individual WPV strains for more than a year has been recently documented in African Region and Eastern Mediterranean Region countries. If AFP surveillance is suboptimal, ongoing poliovirus circulation might not be detected. Certification of wild poliovirus-free status requires at least 3 years of timely and sensitive surveillance (7).

Health systems in the countries most affected by the Ebola outbreak in West Africa (Guinea, Liberia, and Sierra Leone) have been disrupted (8). Although no polio cases have been identified in affected countries, decreases in the national non-polio AFP rates have been noted. As health systems recovery plans are developed, an emphasis on ensuring high quality AFP surveillance, as well as immunization services, will be important.

The primary AFP surveillance quality indicators continued to be met in Afghanistan, Nigeria, and Pakistan in 2014. However, orphan WPV1 and cVDPV2 viruses continue to be identified by genomic sequence analysis in these endemic countries and were also identified in Cameroon, indicating gaps in AFP surveillance. All AFP cases must be identified and reported, and specimens from patients with AFP must be collected and transported appropriately. Environmental surveillance will continue to be an important supplement to AFP surveillance.

The primary surveillance quality indicators do not fully capture any security-related issues, nor the issues associated with mobile and difficult to access populations or other factors that affect surveillance performance. High AFP rates do not necessarily imply sensitive surveillance; anecdotally, visits to some countries have revealed that even in areas meeting surveillance performance indicators, a proportion of the reported AFP cases are unlikely to be true AFP cases. Conversely, evidence from hospital records suggests that some true AFP cases are not being reported. Supervision and monitoring of AFP surveillance may help ensure that all true AFP cases are identified, reported, and investigated appropriately.

As polio case counts decrease, sensitive AFP surveillance becomes increasingly critical. The risk of WPV and cVDPV importation, and cVDPV emergence exists even in countries in polio-free regions. To promptly identify and respond to all cases of polio, surveillance performance must be assessed and quality must be maintained globally.

What is already known on this topic?

Surveillance is a cornerstone of polio eradication efforts. Acute flaccid paralysis (AFP) surveillance is supplemented by environmental surveillance (i.e., the collection of sewage samples for poliovirus testing) in a growing number of countries to identify poliovirus circulation that might occur in the absence of detected AFP cases. The Global Polio Laboratory Network facilitates laboratory identification of polioviruses and provides genomic analysis to help track the spread of both wild and vaccine-derived polioviruses.

**What is added by this report?**

A smaller proportion of World Health Organization African Region countries that had a case of polio since 2010 met the two primary surveillance performance indicators, the non-polio AFP rate and the percentage of stool specimens considered adequate, in 2014 compared with 2013. Surveillance gaps existed at subnational levels. In Ebola-affected countries, polio surveillance quality appears to have decreased.

What are the implications for public health practice?

As polio case counts continue to decrease, sensitive and timely surveillance performance becomes even more critical. Gaps in surveillance quality, especially at the subnational level, must be identified and resolved through well supervised active surveillance, strong passive surveillance, and supplemental environmental and virologic surveillance. As long as polioviruses continue to circulate in any country, all countries remain at risk.

## Figures and Tables

**FIGURE:**
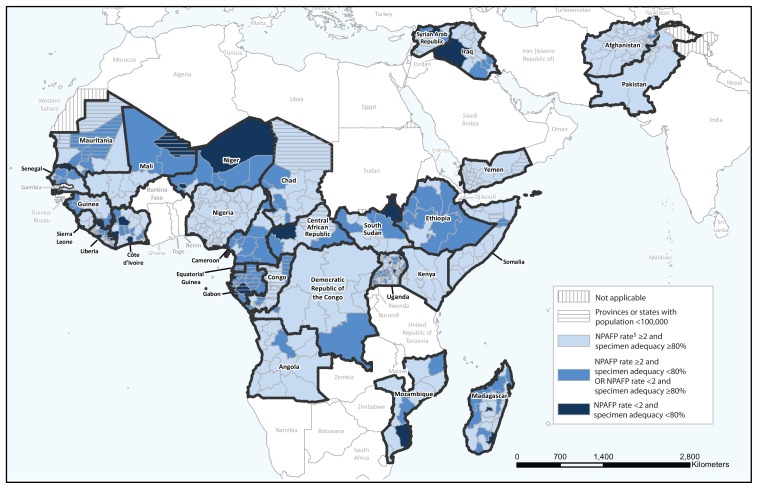
Combined performance indicators for the quality of acute flaccid paralysis (AFP) surveillance* in subnational areas (states and provinces) of 29 countries that were polio-affected during 2010–2014 — World Health Organization African and Eastern Mediterranean regions, 2014^†^ **Abbreviation:** NPAFP = nonpolio AFP. * The Global Polio Eradication Initiative has set the following targets for countries with current or recent wild poliovirus transmission and their states/provinces: 1) NPAFP detection rate of ≥2 cases per 100,000 persons aged <15 years, and 2) adequate stool specimen collection from ≥80% of AFP cases, with specimen adequacy defined as two specimens collected ≥24 hours apart, both within 14 days of paralysis onset, shipped on ice or frozen packs, and arriving in good condition at a World Health Organization–accredited laboratory. ^†^ Data are for AFP cases with onset during 2014, reported as of March 27, 2015. ^§^ Per 100,000 persons aged <15 years.

**TABLE 1:** National and subnational acute flaccid paralysis surveillance indicators and number of confirmed wild poliovirus and circulating vaccine-derived poliovirus cases, by country, including all countries with poliovirus transmission over the past five years (2010–2014) within the two currently polio-endemic World Health Organization regions (African Region and Eastern Mediterranean Region), 2013* and 2014*

2013								

WHO region/Country	AFP cases	Regional/National NPAFP rate†	Subnational areas with NPAFP rate ≥2§ (%)	Regional/National AFP cases with adequate specimens¶ (%)	Subnational areas with ≥80% adequate specimens (%)	Population in areas meeting both indicators** (%)	Confirmed WPV cases*	Confirmed cVDPV cases*††
**AFR**	**20,547**	**5.0**	**—**	**(90)**	—	**—**	**80**	**13**
Angola	310	2.9	(94)	(92)	(94)	(95)	0	0
Cameroon	483	4.3	(100)	(80)	(50)	(47)	4	4
CAR	60	2.6	(57)	(85)	(57)	(23)	0	0
Chad	500	8.6	(100)	(92)	(94)	(91)	0	4
Cote d’Ivoire	455	4.9	(100)	(89)	(83)	(85)	0	0
DRC	2,011	4.8	(100)	(86)	(91)	(91)	0	0
Equatorial Guinea	0	0	(0)	NA	NA	(0)	0	0
Ethiopia	1,164	2.7	(73)	(83)	(55)	(81)	9	0
Gabon	6	0.6	(67)	(50)	(0)	(0)	0	0
Guinea	224	4	(100)	(96)	(100)	(100)	0	0
Kenya	632	3.4	(88)	(83)	(75)	(56)	14	0
Liberia	50	2.9	(80)	(100)	(100)	(86)	0	0
Madagascar	397	4	(90)	(87)	(71)	(67)	0	0
Mali	243	3.1	(88)	(86)	(75)	(79)	0	0
Mauritania	58	4.2	(100)	(91)	(92)	(90)	0	0
Mozambique	323	3.1	(100)	(89)	(80)	(85)	0	0
Niger	338	3.9	(100)	(80)	(63)	(42)	0	1
Nigeria	8,648	10.6	(100)	(96)	(100)	(100)	53	4
Republic of the Congo	106	5.2	(100)	(81)	(64)	(78)	0	0
Senegal	231	3.7	(100)	(73)	(45)	(46)	0	0
Sierra Leone	171	6.4	(75)	(93)	(100)	(79)	0	0
South Sudan	294	3.8	(90)	(94)	(90)	(87)	0	0
Uganda	486	3.3	(72)	(86)	(75)	(52)	0	0
**EMR**	**11,246**	**5.5**	**(82)**	**(90)**	**(78)**	**(73)**	**336**	**53**
Afghanistan	1,897	10.8	(100)	(93)	(97)	(97)	14	3
Iraq	444	3.1	(95)	(84)	(68)	(69)	0	0
Pakistan	4,790	6.0	(88)	(89)	(100)	(99)	93	48
Somalia	546	6.4	(100)	(87)	(79)	(71)	194	1
Syrian Arab Republic§§	180	1.3	(23)	(62)	(31)	(4)	35	0
Yemen	614	5.2	(100)	(91)	(91)	(84)	0	1

2014								

WHO region/Country	AFP cases	Regional/National NPAFP rate†	Subnational areas with NPAFP rate ≥2§ (%)	Regional/National AFP cases with adequate specimens¶ (%)	Subnational areas with ≥80% adequate specimens (%)	Population in areas meeting both indicators** (%)	Confirmed WPV cases*	Confirmed cVDPV cases*††
**AFR**	**22,451**	**5.5**	**—**	**(89)**	**—**	**—**	**17**	**33**
Angola	321	3.1	(100)	(93)	(94)	(97)	0	0
Cameroon	845	8.2	(100)	(72)	(20)	(26)	5	0
CAR	89	4.2	(71)	(78)	(71)	(50)	0	0
Chad	394	7	(100)	(85)	(72)	(73)	0	0
Cote d’Ivoire	395	4.1	(94)	(86)	(61)	(73)	0	0
DRC	1,829	4.6	(100)	(82)	(82)	(76)	0	0
Equatorial Guinea	32	7.8	(100)	(16)	(0)	(0)	5	0
Ethiopia	1,198	2.9	(82)	(76)	(27)	(27)	1	0
Gabon	42	4.8	(78)	(31)	(10)	(0)	0	0
Guinea	146	2.6	(75)	(88)	(89)	(52)	0	0
Kenya	724	4.3	(100)	(88)	(100)	(100)	0	0
Liberia	23	1.2	(58)	(96)	(92)	(21)	0	0
Madagascar	421	4.2	(91)	(84)	(55)	(65)	0	1
Mali	236	3	(100)	(89)	(75)	(92)	0	0
Mauritania	53	3.8	(92)	(81)	(62)	(45)	0	0
Mozambique	317	3	(90)	(87)	(70)	(77)	0	0
Niger	249	2.9	(75)	(71)	(13)	(14)	0	0
Nigeria	10,507	12.9	(100)	(97)	(100)	(100)	6	30
Republic of the Congo	114	5.0	(100)	(87)	(75)	(91)	0	0
Senegal	190	3.3	(82)	(76)	(36)	(48)	0	0
Sierra Leone	71	2.7	(75)	(96)	(100)	(79)	0	0
South Sudan	322	4.2	(70)	(88)	(80)	(64)	0	2
Uganda	576	3.8	(80)	(81)	(58)	(46)	0	0
**EMR**	**12,505**	**6.1**	**—**	**(91)**	**—**	**—**	**342**	**21**
Afghanistan	2,420	13.7	(100)	(92)	(97)	(99)	28	0
Iraq	591	4.1	(89)	(89)	(79)	(74)	2	0
Pakistan	5,327	6.5	(88)	(88)	(100)	(99)	306	21
Somalia	420	8	(100)	(97)	(95)	(99)	5	0
Syria§§	305	3.1	(93)	(82)	(71)	(58)	1	0
Yemen	578	4.9	(100)	(95)	(100)	(100)	0	0

**Abbreviations:** — = not calculated, AFP = acute flaccid paralysis; AFR = African Region; CAR = Central African Republic; cVDPV = circulating vaccine-derived poliovirus; DRC = Democratic Republic of the Congo; EMR = Eastern Mediterranean Region; NA = stool specimens not collected; NPAFP = non-polio AFP; WHO = World Health Organization; WPV = wild poliovirus.

* Data as of March 27, 2015.

† Per 100,000 persons aged <15 years.

§ For all subnational areas regardless of population size.

¶ Standard WHO target is adequate stool specimen collection from ≤80 of AFP cases, in which two specimens are collected ≥24 hours (in this data set this is a treated as ≥1 calendar day) apart, and within 14 days of paralysis onset, and arrive in good condition (received on ice or frozen ice packs, and without leakage or desiccation) in a WHO-accredited laboratory.

** For all subnational areas regardless of population size. The two indicators are 1) National NPAFP rates of ≥2 and 2) ≥80% of AFP cases with adequate specimens (see footnote 4).

†† cVDPV is associated with two or more cases of AFP. Note, however, that the Madagascar event in 2014 occurred in one AFP case and three contacts.

§§ The NPAFP rate for Syria is artificially low because of displaced populations and the lack of official data from areas not under government control.

**TABLE 2:** Number of poliovirus isolates from stool specimens of persons with acute flaccid paralysis and timing of results, by World Health Organization region, 2013* and 2014*

2013								2014						
	
WHO Region	No. of specimens	No. of poliovirus isolates			Poliovirus isolation results on time¶ (%)	ITD results within 7 days** (%)	ITD results within 60 days†† (%)	No. of specimens	No. of poliovirus isolates			Poliovirus isolation results on time¶ (%)	ITD results within 7 days** (%)	ITD results within 60 days†† (%)
	
		Wild	Sabin†	cVDPV§					Wild	Sabin†	cVDPV§			
African	42,316	598	2,861	12	(92)	(88)	(84)	45,856	83	4038	37	(92)	(86)	(92)
Americas	1,672	0	33	0	(80)	(95)	(91)	1,675	0	39	0	(83)	(100)	(94)
Eastern Mediterranean	20,783	125	626	53	(99)	(98)	(97)	23,552	329	809	27	(98)	(95)	(97)
European	3,404	0	37	0	(99)	(93)	(86)	3.224	0	26	2	(99)	—	(82)
South-East Asia	116,179	0	3,274	0	(98)	(91)	(98)	115,539	0	2785	3	(97)	(90)	(98)
Western Pacific	13,304	0	241	0	(65)	(100)	(99)	13,852	0	352	11	(78)	(96)	(81)
**Total** §§	**197,658**	**723**	**7,072**	**65**	**(89)**	**(94)**	**(93)**	**203,698**	**412**	**8,049**	**80**	**(91)**	**(93)**	**(91)**

**Abbreviations:** cVDPV = circulating vaccine-derived poliovirus, ITD = intratypic differentiation.

* Data as of February 25, 2015.

† Either concordant Sabin-like results in ITD test and VDPV screening, or <1% VP1 sequence difference compared with Sabin vaccine virus (<0.6% for type 2).

§ For poliovirus types 1 and 3, 10 or more VP1 nucleotide differences from the respective PV; for PV type 2, six or more VP1 nucleotide differences from Sabin type 2 PV.

¶ Results reported within 14 days for laboratories in the following WHO regions: African, Americas, Eastern Mediterranean, and South-East Asia, and Western Pacific. Results reported within 28 days for the European Region.

** Results of ITD reported within 7 days of receipt of specimen. As EURO performance can be underestimated because of data entry issues, it has been excluded from analysis.

†† Results reported within 60 days of paralysis onset for all WHO regions except Eastern Mediterranean region, which reported within 45 days of paralysis onset.

§§ For last two indicators, total represents the mean of regions’ performance (in %).

## Footnotes

* Genetic clusters consist of WPV isolates with >95% VP1 nucleotide identity.
